# Phosphate sensitivity of KPC-2: a hidden variable in β-lactamase kinetics

**DOI:** 10.1128/aac.01069-25

**Published:** 2026-01-30

**Authors:** Dignite F. Ngango, André Birgy, Timothy Palzkill

**Affiliations:** 1 Verna and Marrs McLean Department of Biochemistry and Molecular Pharmacology, Baylor College of Medicine 3989, Houston, Texas, USA; 2 IAME, UMR 1137, INSERM, Université Paris Cité 555089, Paris, France; University of Fribourg, Fribourg, Switzerland

**Keywords:** beta-lactam antibiotics, enzymology, beta-lactamase, antibiotic resistance

## Abstract

The catalytic activity of β-lactamases, particularly class A enzymes like KPC-2, is central to β-lactam antibiotic resistance. While phosphate buffers are widely used in enzymatic assays due to their physiological relevance, their potential to interfere with enzyme function remains underappreciated. Here, we demonstrate that phosphate acts as a competitive inhibitor of KPC-2 β-lactamase, significantly reducing catalytic efficiency in a concentration-dependent manner. This inhibition is mediated through interactions with threonine 237, as substitution with glycine (T237G) abolishes the inhibitory effect. In contrast, CTX-M-14 and TEM-1, which possess serine and alanine at the same position, respectively, exhibit minimal phosphate sensitivity, underscoring enzyme-specific buffer effects. Structural and kinetic analyses indicate that phosphate binding at the active site primarily impairs substrate affinity and, in some cases, also reduces catalytic turnover. These findings highlight the potential impact of buffer selection in β-lactamase assays and suggest that phosphate-mediated inhibition may lead to underestimation of enzyme activity and inhibitor potency, particularly in studies involving KPC-2. Standardizing assay conditions is essential for accurate evaluation of β-lactamase function and resistance mechanisms.

## INTRODUCTION

Antibiotic resistance remains one of the most pressing global public health challenges, contributing to approximately 10 million deaths annually (1). A major mechanism of resistance is the bacterial production of β-lactamase enzymes that hydrolyze the β-lactam ring found in antibiotics such as penicillins, cephalosporins, and carbapenems (2, 3). This hydrolysis renders the antibiotic ineffective, conferring resistance to a broad spectrum of β-lactam drugs (3). β-lactamases are classified into four major classes (A, B, C, and D) based on their amino acid sequence homology and catalytic properties (4, 5). Class A β-lactamases are widespread, and certain enzymes in the class, such as KPC-2, are particularly concerning due to their ability to confer resistance to last-resort antibiotics such as carbapenems (6–8).

Characterizing β-lactamase activity *in vitro* is crucial to understand their substrate specificity and inhibition profile, which, in turn, is essential for guiding the development or optimization of β-lactam/β-lactamase inhibitor combinations. To characterize *in vitro* enzyme activity, including that of β-lactamase, Michaelis-Menten kinetics are classically employed to obtain the catalytic turnover number (*k*_cat_), the Michaelis-Menten constant (*K*_M_), which is the substrate concentration needed to attain half maximum reaction velocity, and catalytic efficiency (*k*_cat_/*K*_M_) (9). These assays are sensitive to buffer composition, which is important for maintaining optimal pH and enzyme stability. An ideal buffer should have a pKa close to the desired pH of the reaction to provide maximum buffering capacity (10). Since enzymes function optimally within specific pH ranges, buffers with a similar pKa, such as phosphate, HEPES (*N*-2-hydroxyethylpiperazine-N′−2-ethanesulfonic acid), and Tris-HCl (hydroxymethyl aminomethane hydrochloride) are often used interchangeably. Phosphate buffer is frequently preferred due to its physiological relevance and abundance in cellular environments, including the bacterial periplasm (11). Despite their widespread use, buffer components can interact with enzymes and influence catalytic behavior. Phosphate, for example, has been shown to inhibit urease and other metalloenzymes and to interfere with antioxidant inhibition of *Clostridium botulinum* (12–14). Alternatively, phosphate promotes clavulanic acid hydrolysis in BlaC, a class A serine β-lactamase in *Mycobacterium tuberculosis*, with structural studies suggesting that it may act as an alternative nucleophile or facilitate Ser70 reactivation, although the exact mechanism remains unclear (15). More recently, it has been shown that epistasis effects between mutations in the BlaC enzyme are highly buffer-dependent and that the presence of phosphate can alter enzyme activity compared to MES buffer (16). Additionally, multiple studies report variability in enzymatic parameters that depend on the buffer and concentration used, thereby complicating reproducibility across experiments and studies (2). For example, meropenem hydrolysis by KPC-2 shows a 27-fold reduction in catalytic efficiency and a 5-fold increase in *K*_M_ in 50 mM phosphate compared to 10 mM HEPES buffer, suggesting competitive inhibition by phosphate (17, 18). However, the molecular basis of this inhibition and its generalizability across class A β-lactamases remains poorly understood.

**Fig 1:**
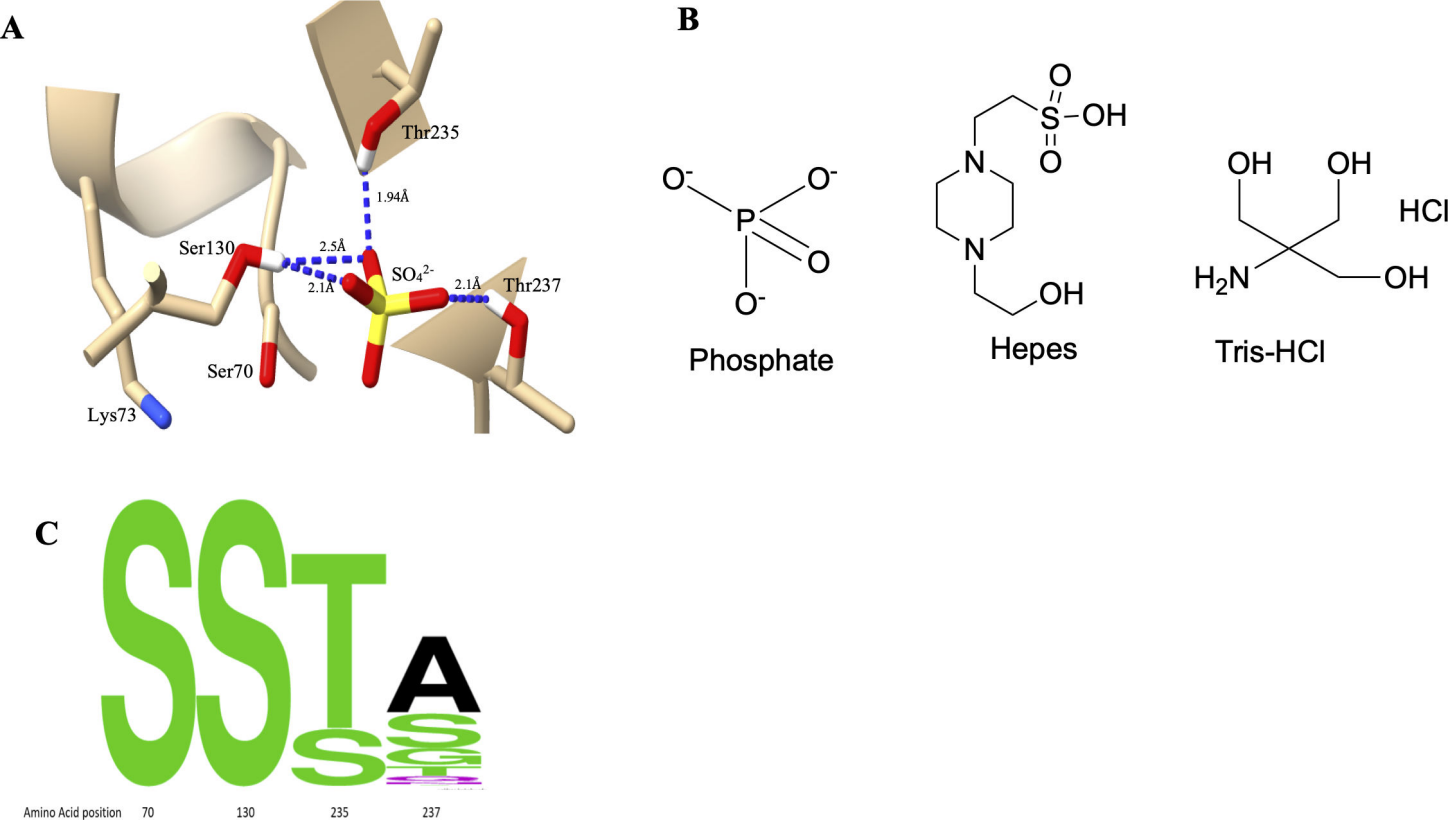
Sulfate binding to the KPC-2 β-lactamase active site and residue variability at positions 70, 130, 235, and 237 in class A β-lactamases. (**A**) Structure of KPC-2 β-lactamase (PDB: 5UL8) showing a sulfate ion hydrogen-bonded (blue dashed lines) to Ser130, Thr235, and Thr237. (**B**) Structures of phosphate, HEPES, and Tris-HCl, highlighting the structural similarity between phosphate and the sulfate group in HEPES. (**C**) Amino acid sequence conservation at positions 70, 130, 235, and 237 based on a structural alignment of 156 class A β-lactamases. Letter size reflects the number of enzymes sharing a given residue. Amino acid variability is highest at position 237 compared to positions 70, 130, and 235.

Examination of 16 structures of KPC β-lactamase in the Protein Data Bank reveals a sulfate ion—whose structure closely resembles that of the phosphate ion—bound in the active site and forming hydrogen bonds (<3 Å) with key catalytic residues such as Ser130, Thr235, and Thr237 (Fig. 1A) (6, 19–22). As sulfate is commonly present in protein purification and crystallization buffers, its recurrent binding at this site suggests that phosphate, due to its structural similarity, may also interact similarly with the active site and influence enzyme activity (Fig. 1B). Furthermore, structures of non-covalent heteroaryl phosphonate inhibitors of KPC-2 β-lactamase show that the phosphate is positioned in a similar position as sulfate in the structures discussed above (23). Structural alignment of 156 class A β-lactamases shows that Thr237 is less conserved than Ser130, Thr235, and Ser70 (Fig. 1C), and prior studies indicate it plays a lesser role in catalysis, suggesting this residue may influence phosphate binding differentially between class A enzymes (2). We therefore hypothesized that phosphate inhibits KPC-2 by forming hydrogen bonds with Ser130, Thr235, and Thr237, and that polarity at position 237 is necessary for this inhibition.

To test this, we monitored cephalothin hydrolysis by four common and representative class A β-lactamases having a different residue at the 237 position: KPC-2 (Thr237) and its T237G mutant, CTX-M-14 (Ser237), and TEM-1 (Ala237), across commonly used buffers. Our results show that phosphate inhibits KPC-2 activity in a concentration-dependent manner, and this effect is mitigated by mutation at position 237. In contrast, CTX-M-14 and TEM-1 exhibited minimal buffer-dependent variation, suggesting that phosphate inhibition is enzyme-specific.

These results emphasize the important role of buffer selection in enzymatic assays, as buffer–enzyme interactions can significantly influence enzyme activity. Understanding these interactions is essential for improving assay reliability and establishing standardized conditions in antibiotic resistance research, which is necessary to ensure that results are comparable across different studies. For example, using phosphate buffer in studies of KPC-2 can lead to underestimation of inhibitor potency due to phosphate-mediated inhibition. Although this study focuses on β-lactamases, the insights are broadly applicable to other enzymes and can guide optimization of assay conditions across systems.

## RESULTS

### Variations in catalytic parameters across commonly used kinetic buffers

To investigate the hypothesis that phosphate inhibits KPC-2 activity and that Thr237 contributes to the inhibition, we performed steady-state kinetic assays for cephalothin hydrolysis using four class A β-lactamases: KPC-2, its T237G mutant, CTX-M-14, and TEM-1. These enzymes were tested across commonly used buffers varying in phosphate concentration, buffering agent (HEPES or Tris), and ionic strength (Tables 1 and 2). KPC-2 showed pronounced buffer-dependent variability with phosphate exhibiting an inhibitory effect. *k*_cat_/*K*_M_ decreased from 5 μM^−1^ s^−1^ in 10 mM PO₄ to 2 μM^−1^ s^−1^ in 50 mM PO₄, with the addition of NaCl partially alleviating this effect. In contrast, KPC-2 displays enhanced catalytic efficiency in HEPES and Tris buffers, with *k*_cat_/*K*_M_ reaching 10 μM^−1^ s^−1^ (fivefold increase compared with 50 mM PO₄) in 10 mM HEPES + 150 mM NaCl and 30 μM^−1^ s^−1^ (15-fold increase compared with 50 mM PO₄) in 50 mM Tris. Moreover, *K*_M_ was 15-fold lower in Tris buffer and 3-fold lower in HEPES buffer compared to 50 mM PO4, supporting the hypothesis that phosphate inhibits KPC-2, possibly by competing with substrate binding (Table 1; Fig. S1).

**TABLE 1:** Steady-state enzyme kinetics for cephalothin hydrolysis by KPC-2 and KPC-2 T237G in commonly used buffers^*a*^

Enzyme	KPC-2			KPC-2 T237G		
	*k*_cat_ (s^−1^)	*K*_M_ (µM)	*k*_cat_/*K*_M_ (µM^−1^s^−1^)	*k*_cat_ (s^−1^)	*K*_M_ (µM)	*k*_cat_/*K*_M_ (µM^−1^s^−1^)
10 mM PO_4_	120 ± 3	25 ± 3	5 ± 1	30 ± 1	17 ± 2	2 ± 0.5
50 mM PO_4_	150 ± 4	86 ± 7	2 ± 0.6	22 ± 2	30 ± 7	0.7 ± 0.3
50 mM PO_4_ 0.15M NaCl	300 ± 10	110 ± 10	3 ± 1	24 ± 1	12 ± 3	2 ± 0.7
10 mM HEPES 0.15M NaCl	390 ± 10	27 ± 4	10 ± 4	30 ± 2	24 ± 6	1 ± 0.5
50 mM Tris-HCl	160 ± 4	5.5 ± 1	30 ± 6	37 ± 2	12 ± 2	3 ± 1

^*a*^ Values are from nonlinear Michaelis–Menten fits (± error) from at least two independent replicates.

Since KPC-2 showed significant phosphate inhibition (Tables S1 and S2), we investigated the role of residue 237 by constructing a T237G mutant, thereby eliminating hydrogen bonding potential at this position. All kinetic parameters (*k*_cat_, *K*_M_, and *k*_cat_/*K*_M_) remained in the same range across all buffer conditions, demonstrating that Thr237 is important for phosphate-dependent inhibition in KPC-2 (Table 1; Tables S1 and S2; Fig. S2).

CTX-M-14 β-lactamase, which has a serine at position 237, showed moderate sensitivity to buffer composition (Table 2). *K*_M_ remained relatively stable across all tested buffers, while *k*_cat_ significantly increased when using HEPES, Tris, or adding NaCl to phosphate, which nearly doubled these parameters (Table 2; Tables S1 and S2; Fig. S3). Meanwhile, *k*_cat_/*K*_M_ remained stable at 10 μM^−1^ s^−1^ across all buffers, with a twofold increase observed in Tris buffer. Since CTX-M-14 also has a polar residue at position 237 but lacks phosphate inhibition, this suggests that phosphate inhibition is enzyme-specific and not solely dependent on residue polarity at position 237.

**TABLE 2:** Steady-state enzyme kinetics for cephalothin hydrolysis by CTX-M-14 and TEM-1 in commonly used buffers^*a*^

Enzyme	CTX-M-14			TEM-1		
	*k*_cat_ (s^−1^)	*K*_M_ (µM)	*k*_cat_/*K*_M_ (µM^−1^s^−1^)	*k*_cat_ (s^−1^)	*K*_M_ (µM)	*k*_cat_/*K*_M_ (µM^−1^s^−1^)
10 mM PO_4_	540 ± 20	57 ± 7	9 ± 3	98 ± 3	190 ± 20	0.5 ± 0.2
50 mM PO_4_	680 ± 20	79 ± 10	9 ± 2	99 ± 4	160 ± 20	0.6 ± 0.2
50 mM PO_4_ 0.15M NaCl	1100 ± 30	110 ± 9	10 ± 3	120 ± 5	180 ± 20	0.7 ± 0.3
10 mM HEPES 0.15M NaCl	1400 ± 50	150 ± 20	10 ± 3	110 ± 6	370 ± 40	0.3 ± 0.2
50 mM Tris-HCl	830 ± 30	56 ± 7	20 ± 5	110 ± 4	150 ± 10	0.7 ± 0.3

^*a*^ Values are from nonlinear Michaelis–Menten fits (± error) from at least two independent replicates.

Similarly, TEM-1, which has alanine at position 237, displayed a comparable trend to CTX-M-14, including the doubling of *K*_M_ in HEPES buffer (370 vs. 160 μM in phosphate buffer). Despite these buffer-dependent changes, TEM-1 exhibited similar catalytic efficiency, with *k*_cat_/*K*_M_ values ranging from 0.3 to 0.7 μM^−1^ s^−1^ across all conditions (Table 2; Tables S1, S2, and Fig. S4).

We extended our analysis to additional β-lactam substrates, including ampicillin, cefotaxime, and imipenem (Tables S3 to S8). KPC-2 efficiently hydrolyzes imipenem and ampicillin, and therefore, these were used as additional substrates for this enzyme. For the hydrolysis of imipenem by KPC-2, *K*_M_ was higher in phosphate compared to HEPES or Tris buffers, suggesting phosphate-mediated inhibition (Tables S3 and S4). With ampicillin as substrate, the *K*_M_ was also higher in phosphate buffer conditions compared to HEPES or Tris buffers (Tables S5 and S6). Ampicillin hydrolysis by the KPC-2 T237G variant, however, showed only modest buffer-dependent differences (Tables S5 and S6). CTX-M-14 efficiently hydrolyzes cefotaxime, and so it was used as an additional substrate for this enzyme (2). CTX-M-14 exhibited similar kinetic parameters across phosphate and Tris buffers for cefotaxime, suggesting modest sensitivity to phosphate (Tables S7 and 8). Overall, these findings indicate that buffer effects are enzyme-specific but follow consistent trends across different β-lactam classes—penicillins (ampicillin), cephalosporins (first-generation: cephalothin; third-generation: cefotaxime), and carbapenems (imipenem)—at least for the enzymes tested. Notably, phosphate inhibited KPC-2 regardless of the substrate, while CTX-M-14, TEM-1, and the T237G mutant exhibited modest effects across all tested conditions.

### Reduced substrate affinity in KPC-2 with increasing phosphate concentration

Our initial results indicated that phosphate inhibition was more pronounced in KPC-2 compared to the other tested enzymes. However, the variability in ionic strength across different buffer conditions complicated interpretation. To control for this, we conducted steady-state kinetic assays with cephalothin as the substrate, maintaining a constant 136 mM ionic strength by supplementing buffers with NaCl (Table 3; Table S9). *k*_cat_ remained relatively stable across different phosphate concentrations, indicating that phosphate does not directly affect catalytic turnover. *K*_M_ increased from 69 μM in 10 mM PO_4_ buffer to 241 μM in 100 mM PO_4_ buffer, suggesting that phosphate inhibits substrate binding. Similarly, *k*_cat_/*K*_M_ decreased from 2.7 to 0.69 μM^−1^s^−1^ with increasing phosphate concentration, further supporting the inhibitory effect of phosphate on KPC-2 activity (Table 3; Table S9 and Fig. S5). To assess whether this inhibition was specific to phosphate, we performed the same experiment using HEPES at varying concentrations (Table 3; Table S9 and Fig. S6). Cephalothin hydrolysis in HEPES showed a moderate rise in *k*_cat_ and *K*_M_, and a modest decrease in *k*_cat_/*K*_M_ at increasing buffer concentrations due to increased *K*_M_.

**TABLE 3:** Steady-state enzyme kinetics for cephalothin hydrolysis by KPC-2 at different phosphate or HEPES concentrations maintaining 136 mM ionic strength constant^*a*^

	Phosphate			HEPES		
Buffer (μM)	*k*_cat_ (s^−1^)	*K*_M_ (µM)	*k*_cat_/*K*_M_ (µM^−1^s^−1^)	*k*_cat_ (s^−1^)	*K*_M_ (µM)	*k*_cat_/*K*_M_ (µM^−1^s^−1^)
10	187 ± 4	69 ± 5	2.7 ± 0.20	186 ± 7	26 ± 5	7.2 ± 1.40
25	172 ± 4	145 ± 9	1.2 ± 0.08	197 ± 9	36 ± 7	5.5 ± 1.09
50	159 ± 5	198 ± 12	0.84 ± 0.05	208 ± 6	58 ± 5	3.6 ± 0.33
75	125 ± 5	189 ± 18	0.66 ± 0.07	245 ± 16	79 ± 15	3.1 ± 0.62
100	167 ± 7	241 ± 23	0.69 ± 0.07	272 ± 12	81 ± 10	3.4 ± 0.44

^*a*^ Values are from nonlinear Michaelis–Menten fits (± error) from at least four independent replicates.

**Fig 2:**
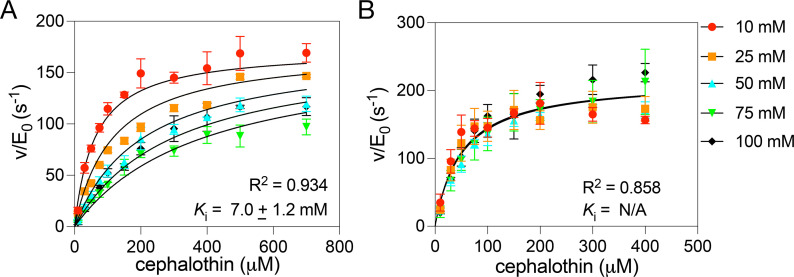
Inhibition profile of KPC-2 β-lactamase with increasing concentrations of phosphate (**A**) or HEPES (**B**) buffer and ionic strength held constant with NaCl. Michaelis-Menten enzyme kinetics analysis was performed at increasing buffer concentrations with cephalothin as substrate. The resulting data were fit globally to a competitive inhibition model. The *R*^2^ and *K*_i_ values are shown at the lower right of each plot. The color code for buffer concentrations is shown at right. Note that the *K*_i_ for HEPES is given as N/A because the fit is unable to provide a confidence interval, and the error is much larger than the value indicating weak, if any, inhibition. Associated errors are from ≥ 4 replicates.

To examine the mode of inhibition of the KPC-2 enzyme by phosphate and potentially by HEPES, we plotted and globally fit the initial velocity data at each cephalothin concentration versus buffer concentration to the competitive inhibition model and the mixed inhibition model (Materials and Methods) (Fig. 2). The initial velocity data in phosphate buffer fit a competitive inhibition model with a *K*_i_ value for phosphate of 7 mM. The data also fit the mixed inhibition model with a similar *R*^2^ value as that for competitive inhibition. The results for competitive inhibition versus mixed inhibition were compared based on the extra-sum-of-squares F-test with competitive inhibition as the null hypothesis, which indicated that the mixed model did not fit significantly better than the competitive model (24). The initial velocity data fit poorly to noncompetitive and uncompetitive inhibition models.

The initial velocity results in HEPES buffer were also fit to a competitive inhibition model, but the *K*_i_ value was >1,000 mM and associated with large error, indicating HEPES is a weak inhibitor of KPC-2 (Fig. 2).

### Inhibition assays confirm specificity and strength of phosphate inhibition

**Fig 3:**
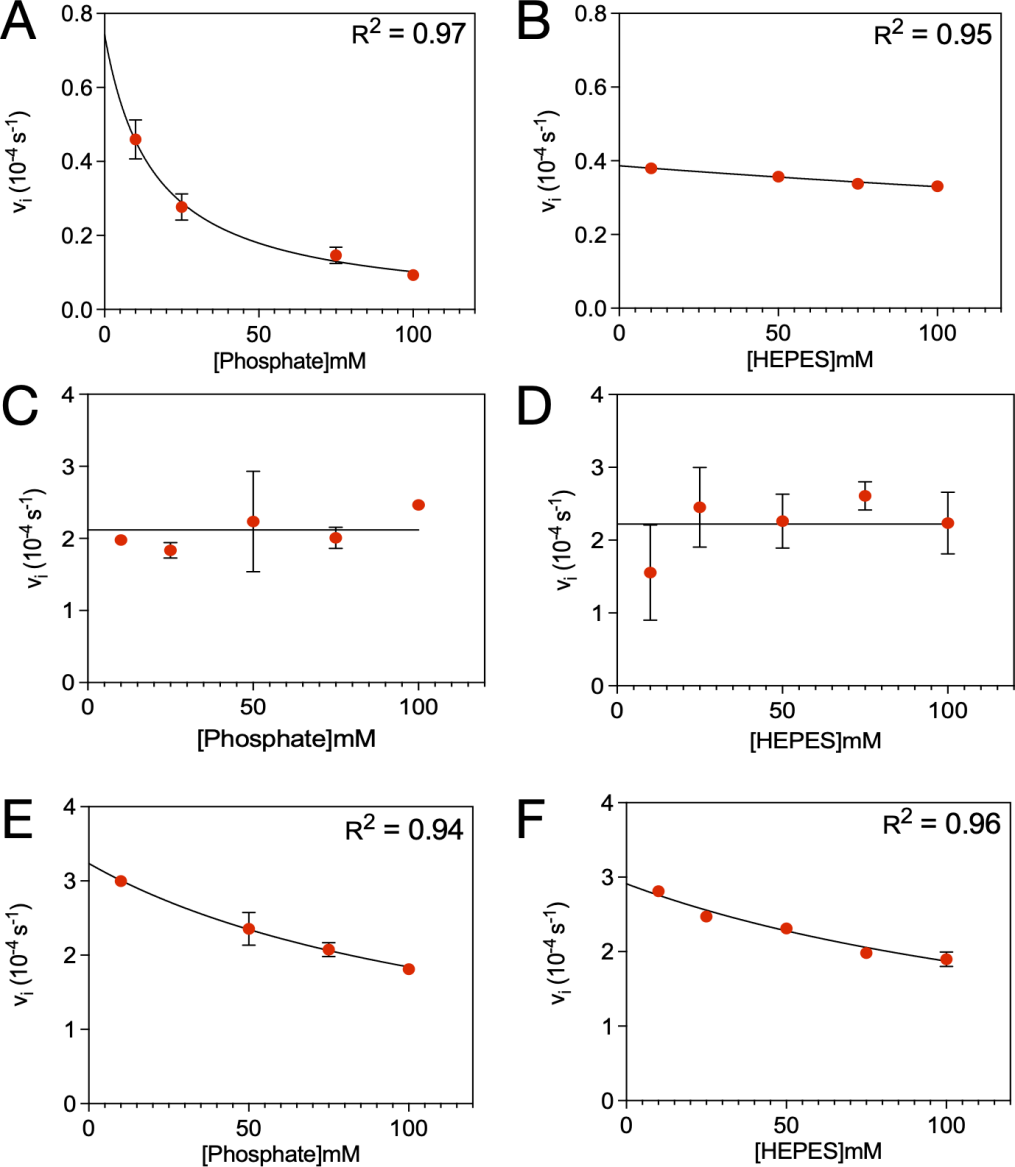
Initial velocities of nitrocefin hydrolysis by KPC-2 (**A and B**), KPC-2 T237G (**C and D**), and CTX-M-14 (**E and F**), as a function of phosphate or HEPES buffer concentration. All reactions were performed at pH 7.0 with an ionic strength of 136 mM and with 50 µM nitrocefin as the reporter substrate. The black lines represent nonlinear regression fits using the Morrison equation to determine the apparent inhibition constant (*K_i_*). Associated errors are from ≥ 2 replicates.

To further quantify buffer-mediated inhibition, we determined the apparent inhibition constants (*K*ᵢ) for phosphate and HEPES using nitrocefin hydrolysis assays (Table 4 and Fig. 3). KPC-2 exhibited inhibition by phosphate with a *K*ᵢ of 7 mM (Fig. 3A), consistent with competitive inhibition observed in kinetic assays (Fig. 2). In contrast, HEPES showed a much weaker inhibitory effect on KPC-2, with a *K*ᵢ of 300 mM—nearly 43-fold higher than phosphate—indicating minimal interference under typical assay conditions (Fig. 3B). This result indicates more potent inhibition of KPC-2 by HEPES than that estimated in steady-state kinetics (Fig. 2), but it is consistent with the overall conclusion that HEPES weakly inhibits KPC-2. To determine whether threonine 237 contributes to phosphate inhibition, the KPC-2 T237G mutant was also tested in the inhibition assay. Notably, the mutant showed no dose-dependent inhibition in either phosphate or HEPES buffer, suggesting that threonine at position 237 is essential for phosphate-mediated inhibition in KPC-2 (Fig. 3C and D).

**TABLE 4:** Inhibition of KPC-2, CTX-M-14, and KPC-2 T237G by phosphate and HEPES^*a*^^,^^*b*^

	*K*_i_ (mM)
Phosphate	
KPC-2	7 ± 2
KPC-2 T237G	ND
CTX-M-14	40 ± 7
HEPES	
KPC-2	300 ± 30
KPC-2 T237G	ND
CTX-M-14	60 ± 7

^*a*^ ND: not determined.

^*b*^ Values are from nonlinear Morrison equation fits (± error) from at least two replicates.

CTX-M-14, which contains a serine at position 237, showed moderate inhibition by both phosphate (*K*ᵢ = 40 mM) and HEPES (*K*ᵢ = 60 mM), suggesting that while polar residues at position 237 may contribute to buffer sensitivity, the magnitude of inhibition is enzyme-specific (Fig. 3E and F).

### Phosphate buffer impacts inhibition of KPC-2 β-lactamase by avibactam and clavulanate

**Fig 4:**
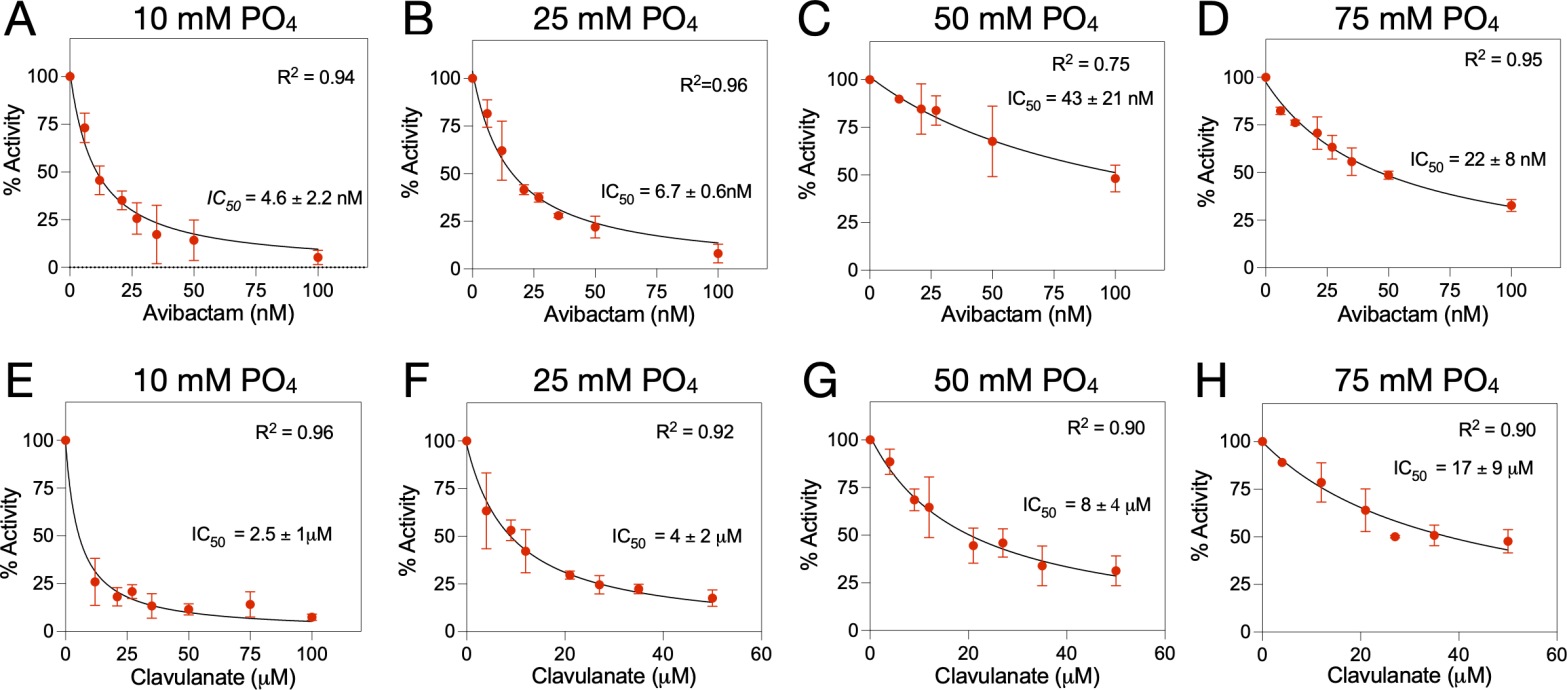
IC_50_ determinations for avibactam and clavulanate for KPC-2 β-lactamase at increasing phosphate buffer concentrations. (**A–D**) IC_50_ results for avibactam in 10, 25, 50, and 75 mM phosphate buffer. (**E–H**) IC_50_ results for clavulanate in 10, 25, 50, and 75 mM phosphate buffer. IC_50_ value, standard error (three independent replicates), and *R*^2^ are shown in each panel.

Finally, we determined whether the concentration of phosphate buffer has an impact on the inhibition potency of KPC-2 by avibactam as indicated by IC_50_ values. The IC_50_ value for avibactam inhibition of KPC-2 was determined in 10 mM, 25 mM, 50 mM, and 75 mM phosphate buffer, where the ionic strength was maintained constant as described for Table 3 above. It was found that the IC_50_ value for avibactam with KPC-2 was 4.6 nM in 10 mM phosphate buffer and increased with increasing phosphate buffer concentration to 22 nM in 75 mM buffer (Fig. 4). Similarly, the IC_50_ value for inhibition of KPC-2 by clavulanic acid increased from 2.5 µM in 10 mM phosphate buffer to 17 µM in 75 mM buffer (Fig. 4). These results suggest phosphate buffer competes with these inhibitors for binding to the enzyme.

## DISCUSSION

This study highlights the significant impact of buffer composition on the catalytic activity of β-lactamases, particularly KPC-2. Our findings suggest that phosphate acts as a competitive inhibitor of KPC-2, likely through interactions with key active-site residues, such as Ser130, Thr235, and Thr237. Structural studies have previously identified sulfate ions interacting with Ser130, Thr235, and Thr237 (6, 19–22). Structural studies have also shown that these residues bind the carboxylate group of β-lactam antibiotics (19, 25). Given the structural similarity between sulfate and phosphate, we hypothesized that phosphate inhibition is a result of similar interactions. Phosphate has also been shown to enhance clavulanic acid hydrolysis in BlaC, though the mechanism remains unclear (15). Moreover, the inconsistent buffer selection across studies has contributed to variability in reported kinetic parameters—even for identical enzyme-substrate pairs. For example, meropenem hydrolysis by KPC-2 shows a 27-fold drop in catalytic efficiency and a 5-fold increase in *K*_M_ in phosphate compared to HEPES buffer (17, 18). These findings emphasize the need to consider buffer composition in enzyme kinetics studies to ensure comparability and reproducibility. In this study, we aimed to investigate the effects of phosphate inhibition, focusing on whether this inhibition is enzyme-specific and identifying residues that may play a role in this process.

We first examined the impact of buffer composition on steady-state kinetics for cephalothin hydrolysis across three class A β-lactamases: KPC-2, CTX-M-14, and TEM-1. While the active site is conserved across these enzymes, residue 237 differs—serine in CTX-M-14, alanine in TEM-1, and threonine in KPC-2 (Fig. 1C). KPC-2 exhibited significant differences in kinetic parameters across buffers, with catalytic efficiency (*k*_cat_/*K*_M_) increasing 5-fold in HEPES and 15-fold in Tris compared to phosphate buffer (Table 1; Tables S1 to S3). This inhibition was concentration-dependent and partially alleviated by NaCl through increased catalytic turnover. The partial alleviation of phosphate inhibition may be related to shielding the negative charge on phosphate by sodium ions and thus reducing interaction with KPC-2 (26).

To investigate whether polarity at position 237 contributes to phosphate inhibition, we generated a T237G mutant of KPC-2, tested TEM-1, which has a nonpolar alanine at this position, and examined CTX-M-14, which has a polar serine. Both the T237G mutant and TEM-1 showed minimal buffer-dependent effects, supporting the idea that hydrogen bonding at position 237 is essential for phosphate inhibition in KPC-2. However, CTX-M-14, which has a polar residue at position 237, did not exhibit comparable inhibition, suggesting that phosphate sensitivity is enzyme-specific and not solely determined by residue polarity.

We further controlled for salt effects by maintaining a constant ionic strength while varying phosphate or HEPES levels (Table 3). Increasing phosphate concentration raised the *K*_M_ (from 69 to 240 µM), consistent with competitive inhibition. HEPES, in contrast, had minimal effect except at high concentrations, possibly due to a similar inhibitory mechanism of its sulfate group. Furthermore, steady-state kinetics analysis at increasing phosphate concentrations showed it competitively inhibits KPC-2 with a *K*_*i*_ of 7 mM, while HEPES is a weak inhibitor of the enzyme (Fig. 2).

To further characterize this inhibition, we performed assays using nitrocefin as a reporter substrate for β-lactamase activity (Table 4 and Fig. 3). KPC-2 exhibited strong phosphate inhibition (*K_i_* = 7 mM), whereas HEPES had a much weaker effect (*K_i_* = 300 mM). The T237G mutant showed no inhibition in either buffer, reinforcing the role of threonine 237 in phosphate binding in KPC-2. Interestingly, CTX-M-14 displayed moderate inhibition by both phosphate (*K_i_* =40 mM) and HEPES (*K_i_* =60 mM), indicating that CTX-M-14 is sensitive to both buffers but with much weaker phosphate inhibition.

These differences may be explained by active-site flexibility and rotamer preferences (Table S10). In CTX-M-14 X-ray structures, Ser237 adopts multiple rotamer conformations, possibly resulting in weaker interactions with phosphate. In contrast, Thr237 in KPC-2 predominantly adopts a rigid gauche conformation, possibly favoring strong phosphate binding (Table S10) (PDB: 5UL8 for KPC-2, PDB:1YLT for CTXM-14).

Finally, we have shown that the presence of increasing phosphate concentration in buffers lowers the apparent potency of inhibition of KPC-2 by avibactam and clavulanate as measured by IC_50_ values, which is again consistent with competition for binding at the active site (Fig. 4).

These findings have significant implications for kinetic assays used to evaluate β-lactamase activity and inhibitors. The strong buffer-dependent variability in KPC-2 underscores the importance of selecting appropriate assay conditions to ensure reliable data. Phosphate, due to its high negative charge, may form strong hydrogen bonds that can interfere with enzyme function, whereas HEPES and Tris, which lack comparable electrostatic interactions, may not exhibit similar inhibition effects. While HEPES contains a sulfate-like moiety that could interact with active-site residues, its bulkier structure may hinder proper alignment in the active site, preventing the sulfate from effectively reaching the binding pocket. Based on our findings, HEPES appears to be a more suitable buffer for accurately assessing KPC-2 kinetics compared to phosphate.

Importantly, phosphate inhibition does not apply universally to all β-lactamases. While threonine 237 plays a key role in KPC-2, other residues may contribute to buffer effects in different enzymes. Since Ser70, Ser130, and Thr235 are conserved and strongly contribute to catalysis, mutating these residues is impractical due to their essential catalytic roles (2). Given that intracellular inorganic phosphate concentrations in bacteria are estimated to be approximately 10 mM, using phosphate buffer to perform kinetics is physiologically relevant (27). However, our data suggest that at *K_i_* = 7 mM, using high concentrations of phosphate—such as the commonly used 50 mM buffer—can interfere with kinetic assays, particularly for KPC-2, potentially leading to underestimation of enzyme activity and inhibitor potency.

Future studies should explore buffer effects across other β-lactamase classes and clinically relevant mutations. Expanding kinetic analyses across diverse β-lactam substrates will help determine the broader applicability of these findings. These insights would aid in the design of optimized assay conditions and improve experimental reproducibility, ultimately enhancing drug development strategies targeting β-lactamase-mediated antibiotic resistance.

## MATERIALS AND METHODS

### Protein expression and purification

KPC-2, KPC-2 T237G, CTX-M-14, and TEM-1 were cloned into the pET28a vector, which includes a His-tag, TEV protease cleavage site for removal of the His-tag, and a kanamycin resistance gene. The proteins were expressed using the *E. coli* BL21 (DE3) system as described previously (28–30). Cultures were grown in LB medium with 25 μg/mL kanamycin at 37°C until reaching an OD_600_ of 0.8. Protein expression was induced with 0.5 mM IPTG, and incubation was continued at 18°C for 16 h before cells were collected by centrifugation.

Cell pellets were resuspended in lysis buffer consisting of either 25 mM sodium phosphate, 300 mM NaCl (for CTX-M-14, TEM-1, and KPC-2 T237G), or 20 mM Tris-HCl, 300 mM NaCl (for wild-type KPC-2), each supplemented with 20 mM imidazole and lysed by sonication. After centrifugation at 10,000 × *g* for 15 min, the soluble fraction was filtered and applied to a metal affinity column (HisTrap FF, GE Healthcare) using an ÄKTA Pure FPLC system. Bound proteins were eluted with a gradient of imidazole (20–500 mM). Eluted fractions were concentrated and buffer-exchanged into the corresponding lysis buffer lacking imidazole.

His-tag removal was performed by incubating the protein overnight at 4°C with TEV protease (1:50 ratio of TEV:protein). The reaction was passed through a nickel resin to remove both TEV protease and any uncleaved β-lactamase. Final purification was achieved through size-exclusion chromatography using a Superdex 75 Increase (10/300) column equilibrated with the corresponding lysis buffer lacking imidazole. Protein purity was assessed by SDS-PAGE, and concentrations were determined spectrophotometrically at 280 nm and using the extinction coefficient as determined by the ExPASy ProtParam tool (31).

### Kinetic analysis of β-lactam hydrolysis

Enzyme kinetic parameters for cephalothin, cefotaxime, ampicillin, and imipenem hydrolysis were determined using a DU800 spectrophotometer. Reactions were monitored at the following wavelengths and extinction coefficients: cephalothin at 262 nm (Δε = 7,660 M⁻¹ cm⁻¹), ampicillin at 235 nm (Δε = 900 M⁻¹ cm⁻¹), cefotaxime at 264 nm (Δε = 7,250 M⁻¹ cm⁻¹), and imipenem at 299 nm (Δε = 9,670 M⁻¹ cm⁻¹). Assays were conducted in multiple buffer conditions to assess the effect of buffer composition on enzyme activity. Reactions were performed at pH 7, with all buffers supplemented with 100 μg/mL bovine serum albumin (BSA) to enhance enzyme stability and minimize adsorption to the cuvette. For each assay, 1 nM of enzyme was mixed with varying concentrations of substrate, and the decrease in absorbance was monitored in real time as substrate hydrolysis progressed. Initial velocities (*v*₀) were determined by fitting the absorbance data using linear regression. These velocities were then plotted against substrate concentration and fitted to the Michaelis–Menten equation (*v* = *V*_max_ [S] / (*K*_M_ + [S])) by non-linear regression to determine the catalytic turnover number (*k*_cat_), Michaelis–Menten constant (*K*_M_), and catalytic efficiency (*k*_cat_/*K*_M_). Data analysis and error estimation were performed using GraphPad Prism.

### Mode of inhibition and *K*_*i*_ determination for phosphate and HEPES inhibition of β-lactamase

All buffers used for experiments in Table 3 and Fig. 3 were adjusted to a final ionic strength of 136 mM using sodium chloride (NaCl), calculated from formula I = ½ Σ (c_i_ z_i_) (2) where I is ionic strength, c_i_ and z_i_ are concentration and valence of ion in the solution, respectively (32). Phosphate buffers were prepared from a 0.1 M stock solution consisting of 42.3% sodium phosphate dibasic (Na_₂_HPO_₄_) and 57.7% sodium phosphate monobasic (NaH_₂_PO_₄_). For final phosphate concentrations of 10, 25, 50, 75, and 100 mM, NaCl was added in the following amounts to achieve 136 mM ionic strength: 123, 102, 68, 34, and 0 mM, respectively. HEPES buffers were prepared using 0.5 M HEPES sodium salt, and for final HEPES concentrations of 10, 25, 50, 75, and 100 mM, NaCl was added at 127, 112, 87, 62, and 37 mM, respectively, to reach the same ionic strength.

 Purified KPC-2 β-lactamase was used for kinetic assays with cephalothin as the substrate. Michaelis–Menten kinetics were determined by measuring the initial rates of cephalothin hydrolysis at varying substrate concentrations (Table 3 and Fig. 3). Reactions were initiated by the addition of enzyme to pre-equilibrated substrate-buffer solutions, and cephalothin hydrolysis was monitored by absorbance changes at 262 nm at 25 °C. A minimum of four replicates of initial velocity determinations were performed at each cephalothin concentration. Kinetic parameters (*k*_cat_ and *K*_M_) were determined by fitting the initial rate data to the Michaelis–Menten equation using GraphPad Prism 10. A minimum of four replicates was performed for each cephalothin concentration for the Michaelis-Menten analysis.

To assess the buffer inhibition effects, the data at different phosphate or HEPES concentrations were each globally fit to both the competitive inhibition model: *v* = (*k*_cat_ * [S])/(*K*_M_ * (1 + [I]/*K*_i_) + [S]) and the mixed inhibition model: *v* = (*k*_cat_ * [S])/(*K*_M_ * (1 + [I]/*K*_i_) + [S] * (1 + [I]/alpha*K*_i_)). The cephalothin substrate concentration [S], the phosphate or HEPES concentration [I], and the *k*_cat_, *K*_M_, and *K*_i_ values were globally fit shared parameters. The global fitting treated the phosphate or HEPES concentration as the inhibitor variable while simultaneously fitting all data sets. The results were compared for the appropriate mode of inhibition with GraphPad Prism using the **“**Compare models” function, and designating the competitive inhibition model as the null hypothesis. The statistical comparison was based on the extra-sum-of-squares F-test and indicated that the null hypothesis could not be rejected (*P* > 0.05), showing the competitive inhibition model provided the best fit of the data for both phosphate and HEPES (24).

### *K_i_* determination for phosphate and HEPES inhibition of β-lactamase

Nitrocefin hydrolysis was monitored using a Tecan plate reader at 482 nm at 28°C. Enzyme concentrations were set at 1 nM for both KPC-2 and the KPC-2 T237G variant, while CTX-M-14 was used at 0.25 nM. Reactions were initiated by adding 50 μM nitrocefin (prepared in water) after enzyme pre-incubation. Assays were performed in varying concentrations of HEPES or phosphate buffers to examine buffer effects, with all buffers adjusted to pH 7 and supplemented with 100 μg/mL BSA to prevent enzyme adsorption. Initial velocities (*v*₀) were determined by fitting the reaction progress curves by linear regression. These velocities were then plotted against buffer concentrations and analyzed using the Morrison equation to determine the inhibition constant (*K_i_*) (33). The Michaelis–Menten constants (*K*_M_) for nitrocefin hydrolysis used were 45, 40, and 25 μM for KPC-2, KPC-2 T237G, and CTX-M-14, respectively. All data analysis and curve fitting were performed using GraphPad Prism (version 10.3.1).

### Determination of avibactam and clavulanate inhibition potency for KPC-2 β-lactamase under varying phosphate concentrations

 The sensitivity of KPC-2 to inhibition by avibactam or clavulanate was assessed using purified enzyme. A total of 2 nM KPC-2 was incubated with varying concentrations of each inhibitor in phosphate buffers of increasing phosphate concentration (Table 3). The ionic strength of the buffers was kept constant with the addition of NaCl, as described above. Incubations were performed at room temperature for 20 min (avibactam) or 10 min (clavulanate). Reactions were initiated by adding 50 µL of the enzyme–inhibitor mixture to 5 µL nitrocefin (50 µM final; prepared from a 1 mM stock in water) and 45 µL of the corresponding buffer, yielding a final volume of 100 µL in a 96-well plate. Nitrocefin hydrolysis was monitored at 28°C using a Tecan plate reader. Initial velocities (*v*_o_) were extracted and normalized to the *v*_0_ obtained in the absence of inhibitor (set to 100%). Percent activity was plotted against inhibitor concentration and fit to the Morrison equation, with *K*_M_ fixed at 40 µM, to obtain IC_50_ values.

## SUPPLEMENTAL MATERIAL

Supplemental Material aac.01069-25-s0001.docx Tables S1 to S10; Fig. S1 to S6.

ASM does not own the copyrights to Supplemental Material that may be linked to, or accessed through, an article. The authors have granted ASM a non-exclusive, world-wide license to publish the Supplemental Material files. Please contact the corresponding author directly for reuse.
