# Supplementary material for: Phosphate sensitivity of KPC-2: a hidden variable in β-lactamase kinetics
Source: Antimicrob Agents Chemother. 2026 Jan 30;70(3):e01069-25. doi: 10.1128/aac.01069-25 (PMC12959137; doi:10.1128/aac.01069-25)
Supplement: Supplemental Material — Tables S1 to S10; Fig. S1 to S6. [file aac.01069-25-s0001.docx]

Supplemental tables

**Table S1**. F-values and associated P values for *k*_cat_ comparisons from Tables 1-2 in the main manuscript, calculated using the extra sum of squares F-test. Each condition was compared to the 50 mM phosphate (PO₄) group as the reference. A significance threshold of p = 0.01 was used and conditions where values are significantly different than the reference are marked*.

| Enzyme | CTX-M-14 | TEM-1 | KPC-2 | KPC-2 T237G |
| --- | --- | --- | --- | --- |
| 10mM PO4 | 26.0 (1, 29); <0.0001* | 0.014 (1, 42); 0.9073 | 38.3 (1, 35); <0.0001* | 5.19 (1, 31); 0.0298 |
| 50mM PO4 | Reference | Reference | Reference | Reference |
| 50mM PO4/0.15mM NaCl | 94.9 (1, 34); <0.0001* | 16.13 (1, 41); 0.0002* | 103 (2, 35); <0.0001* | 0.472 (1, 27); 0.4977 |
| 10mM HEPES/0.15M NaCl | 66.9 (1, 34); <0.0001* | 3.70 (1, 40); 0.0617 | 43.7 (1, 34); <0.0001* | 2.14 (1, 33); 0.1534 |
| 50mM Tris | 14.0 (1, 28); 0.0008* | 0.120 (1, 41); 0.7313 | 0.014 (1, 44); 0.908 | 7.03 (1, 29); 0.0129 |

**Table S2**. F-values and associated P values for K_M_ comparisons from Tables 1-2 in the main manuscript, calculated using the Extra sum of squares F-test. Each condition was compared to the 50 mM phosphate (PO₄) group as the reference. A significance threshold of p = 0.01 was used and conditions where values are significantly different than the reference are marked*.

| Enzyme | CTX-M-14 | TEM-1 | KPC-2 | KPC-2 T237G |
| --- | --- | --- | --- | --- |
| 10mM PO4 | 4.14 (1, 29); 0.0511 | 2.24 (1, 42); 0.1422 | 67.9 (1, 35); <0.0001* | 3.85 (1, 31); 0.0588 |
| 50mM PO4 | Reference | Reference | Reference | Reference |
| 50mM PO4/0.15mM NaCl | 5.34 (1, 34); 0.0271 | 0.850 (1, 41); 0.3621 | 2.75 (1, 35); 0.106 | 5.64 (1, 27); 0.0249 |
| 10mM HEPES/0.15M NaCl | 6.76 (1, 34); 0.0137 | 29.3 (1, 40); <0.0001* | 15.3 (1, 34); 0.0004* | 0.672 (1, 33); 0.6717 |
| 50mM Tris | 3.54 (1, 28); 0.0704 | 4.10 (1, 41); 0.0495 | 121 (1, 44); <0.0001* | 5.17 (1, 29); 0.0306 |

**Table S3**. Steady-state enzyme kinetics for imipenem hydrolysis by KPC-2 β-lactamase in commonly used buffers. Values are from nonlinear Michaelis–Menten fits (± errors) from at least two independent replicates.

| Imipenem | KPC-2 | | |
| --- | --- | --- | --- |
|  | $\boldsymbol{k}_{\boldsymbol{cat}}$ $\boldsymbol{(}\boldsymbol{s}^{\boldsymbol{-1}}\boldsymbol{)}$ | $\boldsymbol{K}_{\boldsymbol{M}}\boldsymbol{(}\boldsymbol{\mu M}^{\boldsymbol{-1}}\boldsymbol{)}$ | $\boldsymbol{k}_{\boldsymbol{cat}}\boldsymbol{/}\boldsymbol{K}_{\boldsymbol{M}}$  $\boldsymbol{(}\boldsymbol{\mu M}^{\boldsymbol{-1}}\boldsymbol{s}^{\boldsymbol{-1}}\boldsymbol{)}$ |
| 10mM PO4 | 36±1 | 110±6 | 0.3±0.1 |
| 50mM PO4 | 39±1 | 290±20 | 0.1±0.05 |
| 50mM PO4  150mM NaCl | 45±2 | 150±20 | 0.3±0.1 |
| 10mM HEPES  150mM NaCl | 24±1 | 48±6 | 0.5±0.1 |
| 50mM Tris HCl | 53±2 | 22±4 | 2±0.6 |

**Table S4**. F-values and associated P values for *k_c_*_at_ and K_M_ comparisons from Table S6 with impenem as substrate, calculated using the extra sum of squares F-test. Each condition was compared to the 10 mM buffer as the reference. A significance threshold of p = 0.01 was used and conditions where values are significantly different than the reference are marked*.

|  | KPC-2 | |
| --- | --- | --- |
| Buffer | *k*_cat_ | *K*_M_ |
| 10mM PO4 | Reference | Reference |
| 50mM PO4 | 4.95 (1, 26); 0.0350 | 122 (1, 26); <0.0001* |
| 50mM PO4 150mM NaCl | 13.5 (1, 26); <0.0011^*^ | 3.57 (1, 26); <0.0700 |
| 10mM HEPES 150mM NaCl | 78.9 (1, 32); <0.0001* | 26.5 (1, 32); <0.0001* |
| 50mM Tris HCl | 20.0 (1, 22);  0.0002* | 27.1 (1, 22);  <0.0001* |

**Table S5**. Steady-state enzyme kinetics for ampicillin hydrolysis by different β-lactamases in commonly used buffers. Values are from nonlinear Michaelis–Menten fits (± errors) from at least two independent replicates.

|  | KPC-2 | | | KPC-2 T237G | | |  |
| --- | --- | --- | --- | --- | --- | --- | --- |
|  | $\boldsymbol{k}_{\boldsymbol{cat}}$ $\boldsymbol{(}\boldsymbol{s}^{\boldsymbol{-1}}\boldsymbol{)}$ | $\boldsymbol{K}_{\boldsymbol{M}}\boldsymbol{(}\boldsymbol{\mu M}^{\boldsymbol{-1}}\boldsymbol{)}$ | $\boldsymbol{k}_{\boldsymbol{cat}}\boldsymbol{/}\boldsymbol{K}_{\boldsymbol{M}}$  $\boldsymbol{(}\boldsymbol{\mu M}^{\boldsymbol{-1}}\boldsymbol{s}^{\boldsymbol{-1}}\boldsymbol{)}$ | $\boldsymbol{k}_{\boldsymbol{cat}}$ $\boldsymbol{(}\boldsymbol{s}^{\boldsymbol{-1}}\boldsymbol{)}$ | $\boldsymbol{K}_{\boldsymbol{M}}\boldsymbol{(}\boldsymbol{\mu M}^{\boldsymbol{-1}}\boldsymbol{)}$ | $\boldsymbol{k}_{\boldsymbol{cat}}\boldsymbol{/}\boldsymbol{K}_{\boldsymbol{M}}$  $\boldsymbol{(}\boldsymbol{\mu M}^{\boldsymbol{-1}}\boldsymbol{s}^{\boldsymbol{-1}}\boldsymbol{)}$ | |
| 10mM PO4 | 160±6 | 140±10 | 1±0.4 | 210±8 | 68±10 | 3±1 | |
| 50mM PO4 | 160±10 | 240±30 | 0.7±0.3 | 240±9 | 97±10 | 2±1 | |
| 50mM PO4  150mM NaCl | 170±20 | 290±80 | 0.6±0.3 | 270±10 | 96±10 | 3±1 | |
| 10mM HEPES  150mM NaCl | 210±8 | 72±9 | 3±1 | 390±30 | 180±30 | 2±1 | |
| 50mM Tris HCl | 170±8 | 56±10 | 3±1 | 250±10 | 64±10 | 4±1 | |

**Table S6**. F-values and associated P values for *k_c_*_at_ and K_M_ comparisons from Table S4 with ampicillin as substrate, calculated using the extra sum of squares F-test. Each condition was compared to the 50 PO4 mM buffer as the reference. A significance threshold of p = 0.01 was used and conditions where values are significantly different than the reference are marked*.

|  | KPC-2 | | KPC-2 T237G | |
| --- | --- | --- | --- | --- |
| Buffer | *k*_cat_ | *K*_M_ | *k_c_*_at_ | *K*_M_ |
| 10mM PO4 | 0.29 (1, 25); 0.5977 | 12.3 (1, 25); 0.0017^*^ | 3.57 (1, 24); 0.0710 | 3.82 (1, 24); 0.0625 |
| 50mM PO4 | Reference | Reference | Reference | Reference |
| 50mM PO4 150mM NaCl | 0.001 (1, 25); 0.9724 | 0.37 (1, 25); 0.5464 | 5.52 (1, 24); 0.0273 | 0.006 (1, 24); 0.9374 |
| 10mM HEPES 150mM NaCl | 4.86 (1, 26); 0.0366 | 27.0 (1, 26); <0.0001* | 21.0( 1, 33); <0.0001* | 4.34 (1, 33); 0.0451 |
| 50mM Tris HCl | 0.20 (1, 24); 0.6557 | 30.7 (1, 24); <0.0001* | 0.70 (1, 24); 0.4118 | 3.94 (1, 24); 0.0588 |

**Table S7**. Steady-state enzyme kinetics for cefotaxime hydrolysis by CTX-M-14 β-lactamase in commonly used buffers. Values are from nonlinear Michaelis–Menten fits (± errors) from at least two independent replicates.

| Cefotaxime | CTXM-14 | | |
| --- | --- | --- | --- |
|  | $\boldsymbol{k}_{\boldsymbol{cat}}$ $\boldsymbol{(}\boldsymbol{s}^{\boldsymbol{-1}}\boldsymbol{)}$ | $\boldsymbol{K}_{\boldsymbol{M}}\boldsymbol{(}\boldsymbol{\mu M}^{\boldsymbol{-1}}\boldsymbol{)}$ | $\boldsymbol{k}_{\boldsymbol{cat}}\boldsymbol{/}\boldsymbol{K}_{\boldsymbol{M}}$  $\boldsymbol{(}\boldsymbol{\mu M}^{\boldsymbol{-1}}\boldsymbol{s}^{\boldsymbol{-1}}\boldsymbol{)}$ |
| 50mM PO4 | 140±4 | 130±10 | 1±0.4 |
| 10mM HEPES  150mM NaCl | 160±4 | 100±10 | 2±0.4 |
| 50mM Tris HCl | 140±2 | 83±5 | 2±0.4 |

**Table S8**. F-values and associated P values for *k_c_*_at_ and K_M_ comparisons for CTX-M-14 from Table S7 with cefotaxime as substrate, calculated using the extra sum of squares F-test. Each condition was compared to the 50 mM PO4 buffer as the reference. A significance threshold of p = 0.01 was used and conditions where values are significantly different than the reference are marked*.

|  | CTXM-14 | |
| --- | --- | --- |
| Buffer | *k*_cat_ | *K*_M_ |
| 50mM PO4 | Reference | Reference |
| 10mM HEPES 150mM NaCl | 11.6 (1, 68); <0.0011* | 1.95 (1, 68); <0.1675 |
| 50mM Tris HCl | 0.13 (1, 62);  0.7197 | 11.0 (1, 62);  <0.0016* |

**Table S9**. F-values and associated P values for *k_c_*_at_ and K_M_ comparisons from Table 3 in the main manuscript, calculated using the extra sum of squares F-test. Each condition was compared to the 10 mM buffer as the reference. A significance threshold of p = 0.01 was used and conditions where values are significantly different than the reference are marked*.

|  | Phosphate | | HEPES | |
| --- | --- | --- | --- | --- |
| Buffer | *k*_cat_ | *K*_M_ | *k_c_*_at_ | *K*_M_ |
| 10mM | Reference | Reference | Reference | Reference |
| 25mM | 1.18 (1, 130); 0.2794 | 26.9 (1, 130); <0.0001* | 0.99 (1, 68); 0.3212 | 2.07 (1, 68); 0.1550 |
| 50mM | 6.73 (1, 162); 0.0104 | 80.2 (1, 162); <0.0001* | 5.08 (1, 68); 0.0275 | 20.1 (1, 68); <0.0001* |
| 75mM | 21.6 (1, 134); <0.0001* | 32.74 (1, 134); <0.0001* | 15.6( 1, 68); 0.0002* | 21.2 (1, 68); <0.0001* |
| 100mM | 1.86 (1, 144); 0.1748 | 74.7 (1, 144); <0.0001* | 46.3 (1, 68); <0.0001* | 34.2 (1, 68); <0.0001* |

**Table S10**. Dunbrack 2010 Backbone-Dependent Rotamer Distributions for Thr237 in KPC-2 (PDB: 5UL8) and Ser237 in CTX-M-14 (PDB: 1YLT)

| Enzyme | Chi angle | Probability |
| --- | --- | --- |
| KPC-2 Thr237 | -58 | 0.8 |
|  | -177 | 0.1 |
|  | 62 | 0.1 |
| CTX-M-14 Ser237 | 66 | 0.5 |
|  | 180 | 0.4 |
|  | -61 | 0.1 |

Supplemental Figures

**Figure S1.** Michaelis-Menten kinetics of 1 nM KPC-2 (0.5nM for Tris-HCl) with cephalothin in various buffers. Reaction rates were fitted using non-linear regression to the Michaelis-Menten equation: v_i_ = *v*_max_[S]/(K_M_ + [S]). Errors represent at 2 two independent replicates.

**Figure S2.** Michaelis-Menten kinetics of 1 nM KPC-2 T237G with cephalothin in various buffers. Reaction rates were fitted using non-linear regression to the Michaelis-Menten equation: v_i_ = v_max_[S]/(K_M_ + [S]). Errors represent at least 2 independent replicates.

**Figure S3**. Michaelis-Menten kinetics of 1 nM CTX-M-14 with cephalothin in various buffers. Reaction rates were fitted using non-linear regression to the Michaelis-Menten equation: v_i_ = v_max_[S]/(K_M_ + [S]). Errors represent at least 2 independent replicates.

**Figure S4**. Michaelis-Menten kinetics of 1 nM TEM-1 with cephalothin in various buffers. Reaction rates were fitted using non-linear regression to the Michaelis-Menten equation: v_i_ = v_max_[S]/(K_M_ + [S]). Errors represent at least 2 independent replicates.

**Figure S5**. Michaelis-Menten kinetics of 1 nM KPC-2 with cephalothin in buffers with varying phosphate concentrations. Ionic strength fixed at 136mM. Reaction rates were fitted using non-linear regression to the Michaelis-Menten equation: v_i_ = v_max_[S]/(K_M_ + [S]). Errors represent at least 4 independent replicates.

**Figure S6**. Michaelis-Menten kinetics of 1 nM KPC-2 with cephalothin in buffers with varying HEPES concentrations. Ionic strength fixed at 136mM. Reaction rates were fitted using non-linear regression to the Michaelis-Menten equation: v_i_ = v_max_[S]/(K_M_ + [S]). Errors represent at least 4 independent replicates.
